# Effect of the Obesity Epidemic on Kidney Transplantation: Obesity Is Independent of Diabetes as a Risk Factor for Adverse Renal Transplant Outcomes

**DOI:** 10.1371/journal.pone.0165712

**Published:** 2016-11-16

**Authors:** Jennifer M. Kwan, Zahraa Hajjiri, Ahmed Metwally, Patricia W. Finn, David L. Perkins

**Affiliations:** 1 Department of Medicine, University of Illinois at Chicago, Chicago, United States of America; 2 Department of Bioengineering, University of Illinois at Chicago, Chicago, United States of America; 3 Department of Surgery, University of Illinois at Chicago, Chicago, United States of America; University of Toledo, UNITED STATES

## Abstract

**Background:**

Obesity is a growing epidemic in most developed countries including the United States resulting in an increased number of obese patients with end-stage renal disease. A previous study has shown that obese patients with end-stage renal disease have a survival benefit with transplantation compared with dialysis. However, due to serious comorbidities, many centers place restrictions on the selection of obese patients for transplantation. Further, due to obese patients having an increased risk of diabetes, it is unclear whether obesity can be an independent risk, independent of diabetes for increasing adverse renal transplant outcomes.

**Methods:**

To investigate the role of obesity in kidney transplantation, we used the Scientific Registry of Transplant Recipients database. After filtering for subjects that had the full set of covariates including age, gender, graft type, ethnicity, diabetes, peripheral vascular disease, dialysis time and time period of transplantation for our analysis, 191,091 subjects were included in the analyses. Using multivariate logistic regression analyses adjusted for covariates we determined whether obesity is an independent risk factor for adverse outcomes such as delayed graft function, acute rejection, urine protein and graft failure. Cox regression modeling was used to determine hazard ratios of graft failure.

**Results:**

Using multivariate model analyses, we found that obese patients have significantly increased risk of adverse transplant outcomes, including delayed graft function, graft failure, urine protein and acute rejection. Cox regression modeling hazard ratios showed that obesity also increased risk of graft failure. Life-table survival curves showed that obesity may be a risk factor independent of diabetes mellitus for a shorter time to graft failure.

**Conclusions:**

A key observation in our study is that the risks for adverse outcome of obesity are progressive with increasing body mass index. Furthermore, pre-obese overweight recipients compared with normal weight recipients also had increased risks of adverse outcomes related to kidney transplantation.

## Introduction

Obesity is increasing worldwide and has become a major epidemic in developed countries [1]. In the U.S., approximately 35% of adults and 17% of children are obese. Obesity is associated with numerous and diverse comorbidities including diabetes mellitus (DM) type II, peripheral vascular disease (PVD), cardiovascular disease (CD), asthma, osteoarthritis, gallbladder disease and some forms of cancer [2, 3]. In addition, in a multivariate analysis, obesity was shown to be an independent risk factor for end stage renal disease (ESRD) with increasing relative risk with increasing body mass index (BMI). This study analyzed age, gender, education, smoking history, cholesterol levels but not diabetes status [4]. Thus, obesity has become a major economic and health burden for the healthcare system and a challenge for kidney transplantation.

Correlating with the obesity epidemic, the number of obese transplant candidates has also been increasing. However, due to the higher risk of complications, obese patients, defined as BMI of ≥30 kg/m^2^, historically have longer wait times for kidney transplantation and develop increased morbidity while on the waitlist [5, 6]. Because of the associated comorbidities and increased risk of adverse outcomes following transplantation, some centers have excluded patients with a high BMI (e.g., ≥35 kg/m^2^) from transplantation. Nevertheless, a report by Gill et al showed that there is a survival benefit for obese patients receiving kidney transplantation compared to dialysis [7]. Thus, developing strategies to manage patients with obesity and ESRD by treating obesity, managing the comorbidities, or understanding potential molecular targets driving adverse risk is crucial.

Among kidney transplant recipients, most studies show that obesity is associated with a higher risk of graft failure and death [8] and in a meta-analysis, increased delayed graft function (DGF) [9]. Obesity is considered a proinflammatory disease, and previous studies have shown that adipocytes and immune cells within adipose tissue produce proinflammatory cytokines including IL6, TNF alpha and IL1 beta [10]. Following transplantation, proinflammatory cytokines may synergize with alloimmunity to increase adverse outcomes. In this study, we investigated the effects of obesity on outcomes in kidney transplantation.

## Research Design and Methods

We analyzed data from the Scientific Registry of Transplant Recipients (SRTR) which includes data on all active and wait-listed transplant candidates and graft recipients in the United States. BMI, as calculated by height over weight measurements recorded at the time of first ESRD treatment (dialysis or preemptive transplantation), was used to stratify patients into categories defined by the World Health Organization as follows: BMI<18.5kg/m^2^ (underweight), 18.5–24.9kg/m^2^ (normal weight), 25.0–29.9kg/m^2^ (overweight), 30.0–34.9kg/m^2^ (class I obesity), 35.0–39.9kg/m^2^ (class II obesity) and 40+kg/m^2^ (class III obesity).

We filtered the SRTR dataset of 200,756 patients that fit our criteria of having data for all variables (age, gender, graft type, ethnicity, PVD, diabetes, year of transplantation, period of follow up, urine protein, acute rejection, dialysis time of <1yr vs ≥1 yr) in our regression and Cox regression hazard ratio modeling analyses. After filtering the dataset to include subjects with valid variables, we identified 191091 recipients that were included in our analyses. Also, we performed a subgroup analysis for 73, 346 recipients of living donors (LD) (S1 Fig). A comparison between the entire cohort and filtered cohort with gender, age, BMI, ethnicity and medical co-morbidities is shown (S1 Fig). The comparison of the entire cohort to the filtered cohort helped ensure reduction of bias or confounders for assessing variables contributing to adverse outcomes. All the demographics were observed at the first follow-up visit after transplantation and the primary outcomes were positive urine protein (as collected on UNOS form from urinalysis), acute rejection before the graft failure, graft failure, and DGF.

The difference across BMI groups was tested by Kruskal-Wallis test and Chi-squared test for continuous and categorical variables, respectively. The data were summarized by median and interquartiles (25^th,^75^th^) for continuous variables, whereas frequency and percentage for categorical variables. We fitted a logistic regression for each outcome and adjusted for selected baseline covariates (logit(P(outcome = 1)) = β_0 intercept_ + β_1 age +_ β_2 gender +_ β_3 graft type +_ β_4 ethnicity +_ β_5 peripheral vascular disease +_ β_6 Diabetes +_ β_7 time period of transplantation (<2000, 2001–2004, 2005–2008, 2009+) +_ β_8 period of follow-up (<1, 1–5, 6–10, 11–15, 16+) +_ β_9 BMI +_ β_10 induction regimen +_ β_11 immune suppression regimen +_ β_12 HLA mismatch +_ β_13 dialysis_. Pre transplant sensitization was not included in the logistic regression analysis due to missing data and significant loss of sample size. Odds ratios and 95% confidence intervals were determined. We used life-table survival curves to describe the time-to-failure data and log-rank test to compare them. A Cox proportional hazard model was performed on the time to graft failure adjusting for the same covariates as in the logistic regression plus urine protein, acute rejection, DGF, cold ischemia time, donor age, donor race and donor BMI. We reported the hazard ratios and their 95% confidence intervals. Because the effect of covariates on graft failure is not constant over time, the hazard ratio is interpreted as an average effect [11]. All these analyses were conducted using SAS (9.3; SAS Institute, Cary, NC) and p-value < 0.05 was considered statistically significant.

## Results

Patient demographics of kidney transplant recipients were stratified by BMI categories (Table 1). Similar to the demographic of patients with ESRD, a majority of the recipients are male (60%) with age ranges between 35–49 (30%) and 50–64 (24%). White recipients represented 57% of those analyzed, and African Americans comprised 23%. The predominant graft type was deceased donor (61%), which encompasses both standard criteria donor (SCD) (51%) and expanded criteria donor (ECD) (10%) grafts, whereas LD grafts represent 38% of transplants. As recipients increased in BMI, there was a significant increase in the percentage of recipients receiving a deceased donor graft. The incidence of type 2 diabetes and coronary artery disease (CAD) was higher with increasing BMI. Similar to previously reported trends, type I diabetics tend to be more lean than their type 2 diabetic counterparts [12]. Our analysis of each age cohort revealed that BMI significantly increased in each age group, with higher obesity frequencies in the 50–64 age cohort. For example, the percentage of obese class II recipients (BMI 35–39.9 kg/m^2^) increases from 12% (18–34 age group), to 33% (35–49 age group), to 44% (50–64 age group) (S2 Fig). Preinduction status, induction status, HLA mismatch and maintenance regimen for all recipients is included (S1 Table). Most recipients had negative crossmatch (98%), with median cold ischemia time of 11–13 hours, and most were on a maintenance regimen of immune suppression 68–70% across all recipients. The number of HLA mismatches was 3–4 across all recipients.

**Table 1:** Patient Characteristics Stratified by BMI. Transplant recipient demographics frequencies were stratified by BMI. Abbreviations: AfAm: African American, AmIndian = American Indian, SCD = standard criteria donor, ECD = expanded criteria donor; Diabetes I = Type I diabetes, Diabetes II = Type II diabetes, CAD = coronary artery disease, PVD = peripheral vascular disease.

	<18.5	18.5–24.9	25–29.9	30–34.9	35–39.9	40+	Total	
	(n = 5 875) (%)	(n = 60 359) (%)	(n = 65 436) (%)	(n = 39 437) (%)	(n = 15 487) (%)	(n = 4 497) (%)	(n = 191091) (%)	
**Age in years**								p<0.0001
18–34	2192 (37.31)	14876 (24.65)	8796 (13.44)	4401 (11.16)	1828 (11.80)	700 (15.57)	32793 (17.16)	
35–49	1796 (30.57)	19988 (33.12)	19799 (30.26)	11829 (29.99)	5043 (32.56)	1624 (36.11)	60079 (31.44)	
50–64	1462 (24.89)	18954 (31.40)	26626 (40.69)	17344 (43.98)	6853 (44.25)	1833 (40.76)	73072 (38.24)	
65+	425 (7.23)	6541 (10.84)	10215 (15.61)	5863 (14.87)	1763 (11.38)	340 (7.56)	25147 (13.16)	
**Gender**								p<0.0001
Female	3629 (61.77)	25985 (43.05)	22100 (33.77)	14750 (37.40)	6962 (44.95)	2419 (53.79)	75845 (39.69)	
Male	2246 (38.23)	34374 (56.95)	43336 (66.23)	24687 (62.60)	8525 (55.05)	2078 (46.21)	115246 (60.31)	
**Ethnicity**								p<0.0001
AfAm	1098 (18.69)	12102 (20.05)	15072 (23.03)	10468 (26.54)	4674 (30.18)	1443 (32.09)	44857 (23.47)	
AmIndian	43 (0.73)	486 (0.81)	685 (1.05)	473 (1.20)	173 (1.12)	42 (0.93)	1902 (1.00)	
Asian	601 (10.23)	4458 (7.39)	2833 (4.33)	902 (2.29)	217 (1.40)	52 (1.16)	9063 (4.74)	
Hawaiian	39 (0.66)	323 (0.54)	250 (0.38)	145 (0.37)	59 (0.38)	23 (0.51)	839 (0.44)	
Hispanic	724 (12.32)	7959 (13.19)	9146 (13.98)	5017 (12.72)	1667 (10.76)	445 (9.90)	24958 (13.06)	
White	3370 (57.36)	35031 (58.04)	37450 (57.23)	22432 (56.88)	8697 (56.16)	2492 (55.41)	109472 (57.29)	
**Donor type**								p<0.0001
SCD	2969 (50.54)	30195 (50.03)	33606 (51.36)	20695 (52.48)	8373 (54.06)	2431 (54.06)	98269 (51.43)	
ECD	379 (6.45)	5299 (8.78)	7341 (11.22)	4423 (11.22)	1614 (10.42)	420 (9.34)	19476 (10.19)	
Living	2527 (43.01)	24865 (41.20)	24489 (37.42)	14319 (36.31)	5500 (35.51)	1646 (36.60)	73346 (38.38)	
**Donor BMI**								p<0.0001
<18.5	438 (8)	3380 (6)	3011 (5)	1399 (4)	491 (3)	141 (4)	8860 (5)	
18.5–24.9	2263 (43)	22166 (41)	23110 (38)	13232 (35)	4976 (34)	1416 (33)	67163 (38)	
25–29.9	1580 (30)	17791 (33)	20737 (34)	12772 (34)	5039 (34)	1433 (34)	59352 (33)	
30–34.9	594 (11)	7416 (14)	9355 (15)	6581 (18)	2763 (19)	800 (19)	27509 (16)	
35–39.9	205 (4)	2242 (4)	2916 (5)	2191 (6)	955 (6)	299 (7)	8808 (5)	
40+	136 (2.6)	1612 (3)	1964 (3)	1221 (3)	561 (4)	179 (4)	5673 (3)	
**Comorbidities**								
Diabetes I	117 (1.99)	2058 (3.41)	2115 (3.23)	1271 (3.22)	482 (3.11)	121 (2.69)	6164 (3.23)	p<0.0001
Diabetes II	209 (3.56)	3335 (5.53)	7695 (11.76)	7452 (18.90)	3624 (23.40)	906 (20.15)	23221 (12.15)	p<0.0001
Stroke	39 (2.38)	467 (2.59)	698 (3.18)	456 (3.21)	171 (2.90)	54 (3.50)	1885 (2.98)	p<0.0001
CAD	27 (2.57)	548 (4.50)	1128 (7.44)	826 (8.20)	336 (8.00)	73 (6.85)	2938 (6.72)	p<0.0001
PVD	41 (2.41)	610 (3.25)	932 (4.06)	755 (5.06)	344 (5.54)	65 (4.02)	2747 (4.15)	p<0.0001
Dialysis	2463 (85.05)	24092 (84.90)	22662 (84.58)	11780 (85.66)	4137 (87.04)	1439 (88.12)	66573 (85.13)	P<0.0001

We analyzed the effect of obesity stratified by BMI on adverse outcomes including delayed graft function (DGF), acute rejection (AR), positive urine protein, and graft failure in kidney transplant recipients (Table 2). Based on Chi-square analysis, BMI had a significant association with incidence of all 4 adverse outcomes in the all recipients group, which includes deceased donor plus LD grafts (Table 2, top, p < 0.001 for all outcomes). For example, the frequency of DGF increased from 12.8% in normal weight recipients to 26.3% in recipients with BMI >40. Similarly, we observed increases in AR from 8.5% to 12.4%, positive urine protein from 35% to 41.2% and graft failure from 16% to 20.4% from normal weight to obese category II. We also performed a subset analysis of LD grafts, and although the overall incidence of adverse outcomes was lower for all types of donors, we again observed significantly more adverse outcomes with higher BMIs except for graft failure, which was not significant. Using the Cochran-Armitage trend test, we accessed whether each adverse outcome increases with increasing BMI. Indeed, all the adverse outcome rates showed a positive association with a higher BMI, except for graft failure (Table 2, bottom).

**Table 2:** Adverse Outcomes Stratified by BMI. The number and percentage of transplant recipient outcomes (DGF, acute rejection, urine protein and graft failure) stratified by BMI and assessed for significant differences across BMI categories by chi square using SAS 9.3. Outcomes of all kidney donor types (deceased and living) are shown on top, and outcomes of living kidney donors shown on bottom. p<0.05 is significant.

All Recipients								
	Total	<18.5	18.5–24.9	25–29.9	30–34.9	35–39.9	40+	
Outcome	(n = 191091) (%)	(n = 5875) (%)	(n = 60359) (%)	(n = 65436) (%)	(n = 39437) (%)	(n = 15487) (%)	(n = 4497) (%)	p value
DGF	31331 (16.26)	658 (11.07)	7770 (12.75)	10561 (16.02)	7649 (19.25)	3498 (22.41)	1195 (26.35)	< .0001
Acute rejection	483 (8.22)	5148 (8.53)	5894 (9.01)	3971 (10.07)	1766 (11.40)	559 (12.43)	17821 (9.33)	< .0001
Urine protein	1825 (31.06)	19596 (32.47)	22920 (35.03)	14538 (36.86)	5967 (38.53)	1856 (41.27)	66702 (34.91)	< .0001
Graft failure	1128 (19.20)	10614 (17.58)	10541 (16.11)	6507 (16.50)	2720 (17.56)	917 (20.39)	32427 (16.97)	< .0001
Recipients of Living Donors								
	Total	<18.5	18.5–24.9	25–29.9	30–34.9	35–39.9	40+	
Outcome	(n = 73 346) (%)	(n = 2 527) (%)	(n = 24865) (%)	(n = 24489) (%)	(n = 14319) (%)	(n = 5500) (%)	(n = 1658) (%)	p value
DGF	2884 (3.93)	76 (3.01)	795 (3.20)	891 (3.64)	656 (4.58)	343 (6.24)	123 (7.47)	< .0001
Acute rejection	6699 (9.13)	224 (8.86)	2085 (8.39)	2129 (8.69)	1431 (9.99)	623 (11.33)	207 (12.58)	< .0001
Urine protein	26464 (36.08)	796 (31.50)	8256 (33.20)	8906 (36.37)	5524 (38.58)	2235 (40.64)	747 (45.38)	< .0001
Graft failure	10219 (13.93)	443 (17.53)	3726 (14.98)	3132 (12.79)	1829 (12.77)	805 (14.64)	284 (17.25)	< .0001

To determine if demographic factors or comorbidities contributed to the higher incidence of adverse outcomes observed with increasing BMI, we performed a logistic regression analysis analyzing outcomes with covariates including BMI, ethnicity, gender, age, graft type, DM, PVD and stroke (Table 3). The multivariate logistic regression analysis adjusted risk of DGF, AR, positive urine protein and graft failure was significantly increased for all BMI classes (I, II and III) (p < 0.001). The odds ratio (OR) for each adverse outcome, including DGF, acute rejection and urine protein, progressively increased with increasing BMI class. Interestingly, the increased risk also extended to the pre-obese overweight recipients (BMI 25.0–25.9 kg/m^2^) who also had a significantly increased risk for DGF, AR and positive urine protein. (Table 3A) By Cox regression modeling, the hazard ratio was significantly increased for all obese cohorts for graft failure (Table 3B). In summary, the risk of DGF, AR, positive urine protein and graft failure progressively increased with increasing BMI classes. For example, the OR increased from BMI class I to III for DGF (1.47 to 2.43), AR (1.14 to 1.26), urine protein (1.48 to 2.43) and the HR increased for graft failure (1.02 to 1.25), all of which had significant p values (Table 3).

**Table 3:** Adverse Outcomes Relative to Normal BMI. Adverse outcomes of DGF, AR, urine protein relative to normal BMI were calculated using logistic regression analysis. The logistic regression model is adjusted for ethnicity, gender, age, graft type, comorbidities (e.g., diabetes). Odds ratio (OR) outcomes and 95% Confidence intervals (CI) are featured. p<0.05 is significant.

**A**	DGF	OR of having DGF relative to 18.5–24.9			
	BMI	OR	95% CI		p value
	18.5–24.9	1			
	<18.5	0.932	0.854	1.018	0.012
	25.0–29.9	1.188	1.149	1.228	< .0001
	30.0–34.9	1.477	1.423	1.532	< .0001
	35.0–39.9	1.831	1.745	1.922	< .0001
	40+	2.425	2.247	2.616	< .0001
	Acute Rejection	OR of having acute rejection relative to 18.5–24.9			
	BMI	OR	95% CI		p value
	18.5–24.9	1			
	<18.5	0.861	0.775	0.956	0.005
	25.0–29.9	1.078	1.033	1.125	0.0006
	30.0–34.9	1.138	1.084	1.193	< .0001
	35.0–39.9	1.194	1.121	1.271	< .0001
	40+	1.262	1.14	1.397	< .0001
	Urine Protein	OR of having positive urine protein relative to 18.5–24.9			
	BMI	OR	95% CI		p value
	18.5–24.9	1			
	<18.5	0.94	0.867	0.984	0.013
	25.0–29.9	1.19	1.028	1.083	< .0001
	30.0–34.9	1.48	1.04	1.103	< .0001
	35.0–39.9	1.83	1.033	1.119	0.0004
	40+	2.43	1.123	1.285	< .0001
**B**	Cox model Hazard Ratio of Graft Failure relative to 18.5–24.9				
	BMI	p value	HR	95% CI	
	18.5–24.9		1		
	<18.5	0.998	1	0.925	1.047
	25–29.9	0.4156	1.015	0.983	1.047
	30–34.9	< .0001	1.104	1.065	1.145
	35–39.9	< .0001	1.216	1.158	1.276
	40+	< .0001	1.248	1.156	1.348

**Fig 1:**
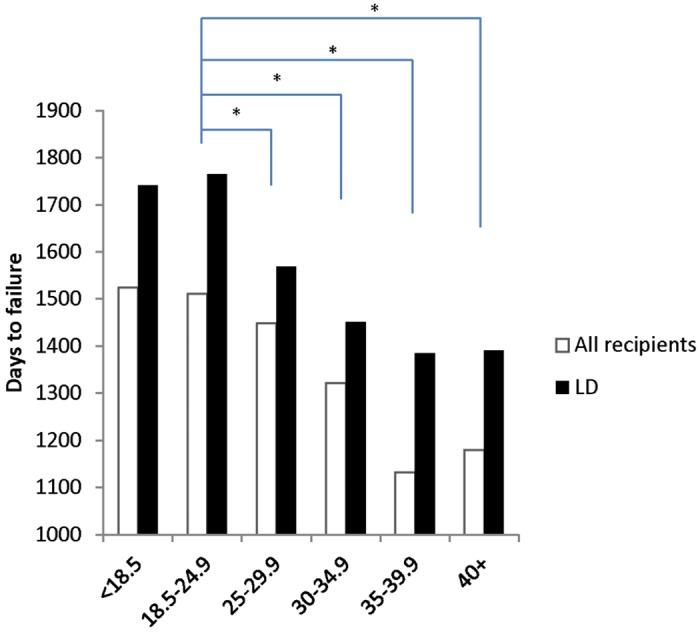
Time to Graft Failure Stratified by BMI. Median Time to graft failure for each BMI category was determined by analyzing the time difference (in days) between date of graft failure and date of transplant. Living donors (black bars), all donors (white bars). p-value < 0.05 is statistically significant.

Next, we assessed whether obesity impacted time to graft failure visually. Obese classes I, II and III all have a significantly shorter time to graft failure relative to normal weight cohorts (Fig 1). For example, obese class I, II and III recipients progressed to graft failure 189, 379 and 331 days quicker, respectively, than normal weight recipients. Interestingly, the overweight recipients also had a shorter time to graft failure than the normal weight group. In a subset analysis of the LD group, although the overall times to graft failure were longer than in the AD groups, we detected significantly shorter times to graft failure with increasing BMI. Specifically, increasing BMI shortens time to graft failure to 1443 days for all obese BMI cohorts compared to 1521 days for normal weight. In an analysis of BMI classes, time to graft failure was 1322 days for obese class I, 1132 days for class II and 1180 for class III. Similar decreases in time to failure were noted in the LD cohort sub-analysis.

**Fig 2:**
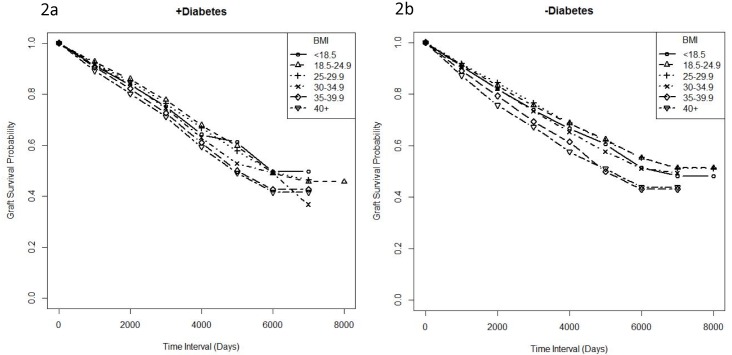
Effect of BMI on Graft Failure is Independent of Diabetes Mellitus. Life-table survival curves were plotted to describe the median time-to-failure for all donor types in recipients with diabetes (Fig 2a) or without diabetes (Fig 2b) for each BMI category and log-rank tests were used to compare them. Time to failure spans 0 was restricted to (time of transplant) to 8000 days (21.9 years). p<0.05 is significant. With calculated p<0.0001 for both +/- diabetes cohorts.

Because there is a strong correlation between obesity and DM, and because DM is a comorbidity for adverse outcomes in transplantation, we used life-table survival curves to describe time to graft failure for each BMI category with or without DM visually. As expected, recipients with DM with a higher BMI had a shorter time to graft failure compared to recipients without DM for each BMI category (Fig 2a). Importantly, we also found that obese transplant recipients without diabetes have a shorter time to failure with increasing BMI (Fig 2b). Thus, BMI is associated with shorter time to graft failure, independent of DM. Similar trends were found in the analysis of the LD subgroups (S3 Fig).

**Fig 3:**
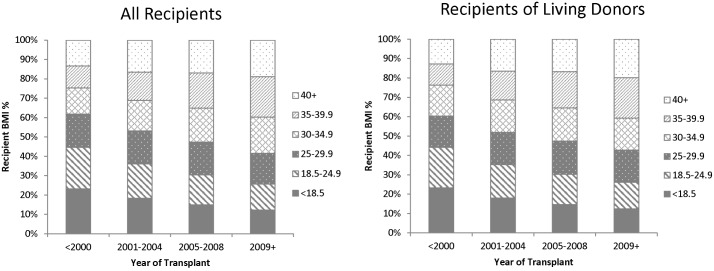
Increasing BMI of Transplant Recipients by Time Period. Temporal trends of BMI in transplant recipients stratified by BMI (<18.5, 18.5–24.9, 25–29.9, 30–34.9, 35–39.9, 40+) for each time period of transplantation (<2000, 2001–2004, 2005–2008, 2009+) tabulated for all donors (left) and living donors (right). Significance across BMI for each time period of transplantation was determined by chi square. p<0.05 is significant.

Obesity is a growing epidemic in the general population so we queried whether the frequency of obese patients receiving transplants was also increasing. Recipients were stratified by BMI and period of transplant dating from 1987 to 2013. Time period categories were binned into the groups: <2000, 2001–2004, 2005–2008 and >2009. Consistent with reported trends [13, 14], analysis of SRTR data found an increase in obese categories of patients being transplanted in recipients of both AD and LD from 1987 to 2013 (Fig 3). For example, the percent of transplants for obese class I, II and III increased from 16% to 31%, 14% to 35% and 17% to 32%, respectively, from <2000 to >2009. As expected, transplants in normal weight recipients amongst all donor types showed a reciprocal decrease.

**Fig 4:**
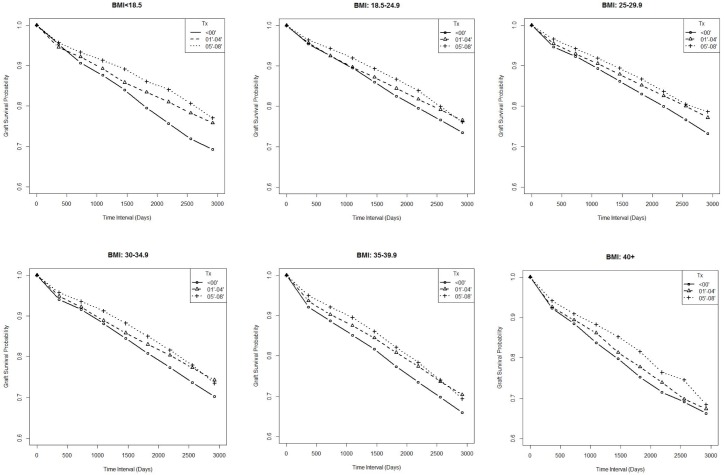
Graft Survival Stratified by BMI for Time Periods <2000, 2001–2004, 2005–2008. Survival rates are shown for 3000 days for each time period of transplantation (<2000, 2001–2004, 2005–2008) stratified for each BMI category.

Next, we asked if graft survival was improving for recipients classified per BMI class over the time periods of the SRTR data (Fig 4 and S4 Fig). Analysis of the SRTR data shows that for each BMI, class survival time has improved from <2000 to 2005–2008 in terms of 4-year survival rates. And as expected, recipients with higher BMIs had decreased survival time for all 3 time periods compared to normal weight counterparts (S5 Fig). For example, analysis of graft survival at 4 years (1460 days) showed increased survival for BMI class I (84.5, 85.8, 88.2 days), class II (81.8, 84.5 and 86.1 days) and class III (80, 81.4 and 85.2 days) for the time periods <2000, 2001–2004 and 2005–2008, respectively. We also detected increases in survival for the normal weight recipients, 86.1 < 87.9 < 89.4, in increased survival time (days). Thus, we detected increases in graft survival at 4 years following transplantation for all 3 BMI classes, as well as normal weight recipients.

## Discussion

In our study, we analyzed 191,091 patients, filtered from 200, 756 kidney transplant patients in the Scientific Registry of Transplant Recipients (SRTR) database from 1987–2013 to determine the effect of obesity on outcomes following transplantation. Patients with complete data points for the variables being assessed were included. Our study shows that the number of obese transplant patients is increasing and that recipients have an increased incidence of adverse outcomes and a shorter time to graft failure. Using multivariate analysis, we showed that obesity is an independent risk factor for adverse transplant outcomes, independent of comorbidities including DM. Importantly, we demonstrate that the risk of adverse outcomes following transplantation progressively increased with higher BMI categories. Interestingly, we also show that the increased risk of adverse outcomes is observed in the pre-obese overweight category for DGF, acute rejection, positive urine protein and marginally significant for graft failure. Due to increases in the obese population, the number of obese patients with ESRD has also been increasing resulting in a greater number of patients on the transplant waiting list. Despite restrictions on the criteria for acceptance of obese patients for transplantation by some centers, the number of obese patients receiving a kidney transplant has also been markedly increasing. As prior work suggest that there has been an increase in the numbers of obese recipients, [13] we found similar trends (Fig 3). The percentage of obese class II recipients has increased during the study period (1987–2013). Similar increases were observed in other obese classes with a corresponding decrease in the percentage of normal weight recipients. These trends were observed in both the AD and LDs categories. Obese donor BMI has been shown to increase the risk of DGF, [15] while another study showed that there was little influence on transplant outcomes unless donor BMI was greater than 45 kg/m.^2^ In our model, we adjusted for donor BMI and still found that recipient BMI had an independent impact on outcomes. [16]

Due to the serious comorbidities of obesity, some centers consider obese patients high risk and establish exclusion criteria for transplantation eligibility. For example, some transplant centers reject candidates with a BMI >35 kg/m^2^. On the other hand, a recent report showed that obese patients have a survival advantage with transplantation compared to dialysis [7]. Thus, obesity is a crucial factor in the selection and management of patients for clinical transplantation. In this study, the large dataset provided high statistical power to detect significant effects of obesity in the transplant recipient population. Our analysis of adverse outcomes including DGF, AR, positive urine protein and graft failure showed increased incidence of all adverse outcomes in all classes of obesity adjusting for the recipients of living and all donor grafts.

Due to the multiple comorbidities of obesity, we performed logistic regression analysis investigating the covariates of age, gender, graft type, ethnicity, PVD, diabetes, time period of transplantation, graft follow up period, urine protein, acute rejection and DGF. The multivariate analysis showed that obesity had an effect on outcomes independent of the covariates. Also, since DM is a major comorbidity of obesity, we generated life survival tables and Cox model for the time to failure for each BMI category with and without DM. The results showed that with increasing BMI graft survival decreased independently of DM. As expected, patients with DM had a trend toward shorter graft survival (although this is not statistically significant in the Cox model) but also had an association with worsening survival with increasing BMI. While not all potential confounding factors are collected in the SRTR dataset, these results indicate that the increase in adverse outcomes may not simply be due to DM or one of the other covariates, but are associated with obesity.

Although acute rejection rates have significantly decreased over the past two decades correlating with the introduction of new therapeutic agents [17, 18], the graft survival times have not markedly improved to reflect the corresponding decrease in acute rejection [19]. We considered that the modest improvement in graft survival could be attributed, at least in part, to the negative effects of the increased proportion of high-risk obese recipients modulating the positive effects of improved medical management. Using a model that analyzed the effects of a single variable, BMI, we estimated that the time to graft failure would be predicted to decrease from 1390 days in the <2000 group to 1323 days in the >2009 group. However, graft survival has modestly improved and not decreased. We favor the interpretation that some factors have improved survival (e.g., immunosuppressive modalities and medical management) while other factors have worsened survival (e.g., increased percentage of high risk patients including obese patients plus older and ECD donors) with a net effect of a small increase in overall survival.

Despite the development of new therapeutic modalities during the past two decades and a concomitant decrease in the incidence of AR, which has been considered a major risk factor for graft survival, overall graft survival has shown only a modest improvement [19]. One possibility is that the increasing percentage of transplant recipients with a high BMI, as illustrated in Fig 3, could be exerting a negative effect on overall transplant outcomes. We illustrate the potential effect of changes in this single factor, BMI, over the time periods of the SRTR data (<2000, 2001–2004, 2005–2008 and >2009) on the time to graft failure (S5 Fig). Due to the increasing percentage of transplant recipients with a high BMI during this time period, our plot suggests that the time to graft failure for all recipients would markedly decrease during the time period of our analyses (<2000 to >2009). However, the actual observed time to graft failure has modestly improved and not worsened as suggested by our BMI model, we suggest that concurrent factors in addition to BMI, such as improved therapeutic modalities and better medical management have contributed to improve outcomes. The net effect of the negative impact of transplanting a high percentage of patients with a high BMI in parallel with the positive impact of improved treatment modalities has been a modest improvement in outcomes.

Several limitations exist for retrospective cohort studies using data entered into a national registry database. These include variability, bias and accuracy of data entry. Further, our study looked at the BMI at time of transplant and was not able to assess the effects of weight gain or loss on outcomes due to the lack of data points.

A key observation in our study is that the risks of obesity are progressive with increasing BMI from class I to III. Interestingly, we also detected increased risk in our overweight patients (BMI 25–29.9 kg/m^2^) compared to normal weight recipients for urine protein, delayed graft function and acute rejection. Thus, there is a graduated risk increasing from the overweight through the obese categories, suggesting that even modest weight loss could provide benefits in terms of outcomes following transplantation. Interestingly, and paradoxically, by the logistic regression model, overweight did not significantly increase risk of graft failure, even though there is a trend toward increased risk. This is consistent with prior reports of overweight being protective in terms of mortality for hemodialysis patients [20, 21]. the mechanisms are unclear but adipose is known to secrete growth and pro survival factors [2, 22]. But once the adipose gets to obese state, it shifts toward a proinflammatory profile that can worsen graft survival [23]. This interpretation is consistent with other studies of weight loss showing that a 5% reduction in weight produces improvement in overall health outcomes for obese cohorts [24, 25]. However, this has yet to be shown for obese renal transplant recipients. Notably, our analysis suggests that the treatment of obesity, could exert improvements in overall transplant outcomes. Protocols of exercise plus diet can induce modest weight loss in some patients [26]. For patients with more extreme requirements for weight loss, bariatric surgery is under investigation in some studies of transplant patients. For example, an ongoing study of robotic surgery is currently investigating transplantation alone or in combination with gastric sleeve. In addition, novel therapies may be required. As registered on ClinicalTrials.gov, there are currently 5723 studies of obesity and 48 studies of obesity in transplantation. Results of these studies will be important to identify the optimal management of the obese transplant patient. In summary, although a prior study suggests that obese patients derive a survival benefit, we show that they have significantly increased risk for several adverse transplant outcomes including delayed graft function, acute rejection and graft failure. Having higher levels of adipose tissue particularly in the abdominal area has been shown to promote a systemic proinflammatory state where both the humoral and cellular immune components work synergistically to promote inflammation [27–29] [30] [31]. Some possibilities from the current literature suggests antagonism of predominantly elevated proinflammatory cytokines such as TNFa, IL6, MCP1, IL2 can be explored as possible ways for immune modulation to decrease systemic inflammation. In terms of further research, systematic approaches examining other factors including immunity, adipose tissue and interplay with kidney function may provide insights into specific molecular targets to improve outcomes for obese renal transplant patients.

## Supporting Information

S1 Fig Data filtering algorithm from entire SRTR dataset. 200756 patients were filtered to identify the study sample, which included pts that had complete data or noted variables. Patients with missing data were excluded. From the patients with complete data, living donor recipients were identified for comparisons to all donors.
(TIF)

S2 Fig BMI stratified by age groups 18–34, 35–49, 50–64 and 65+. (TIF)

S3 Fig Time to failure as stratified by BMI and diabetes amongst living donor subgroup. Life-table survival curves were plotted to describe the time-to-failure of those with diabetes (+diabetes) and those without diabetes (-Diabetes) for each BMI category of the living donor recipient cohort. time to failure was restricted over a time course spanning 0 (time of transplant) to 8000 days (21.9 years) with renal transplant patients stratified by BMI is shown.
(TIF)

S4 Fig Time to failure for each BMI category stratified by time of period of transplantation (<2000, 2001–2004, 2005–2008). Life-table survival curves were plotted to describe the time-to-failure for each BMI category for all recipients for each indicated time period of transplantation. p<0.05 is significant.
(TIF)

S5 Fig Projected impact of increasing number of recipients with high BMI on time to failure in model with all other variables held constant. Using the actual number of recipients for each BMI class for each time period (<2000, 2001–2004, 2005–2008, 2009+), we projected that time to graft failure assuming all other variables were constant.
(TIF)

S1 Table Preinduction status, induction status, HLA mismatch and maintenance regimen for all recipients. (TIF)

## Abbreviations

AD: All Donors including Living and Deceased Donor
AR: Acute Rejection
BMI: Body Mass Index
CAD: Coronary Artery Disease
CI: Confidence Interval
DGF: Delayed Graft Function
DM: Diabetes Mellitus
ESRD: End Stage Renal Disease
ECD: Expanded Criteria Donor
kg: Kilograms
LD: Living Donor
IL1: Interleukin 1
IL6: Interleukin 6
OR: Odds Ratio
PVD: Peripheral Vascular Disease
SCD: Standard Criteria Donor
TNF: Tumor Necrosis Factor

## References

[1] CDC. Adult Obesity Facts 2014 [cited 2014 11/29/14]. Available from: http://www.cdc.gov/obesity/data/adult.html.

[2] Zoccali C, Tripepi G, Cambareri F, Catalano F, Finocchiaro P, Cutrupi S, et al Adipose tissue cytokines, insulin sensitivity, inflammation, and cardiovascular outcomes in end-stage renal disease patients. Journal of renal nutrition: the official journal of the Council on Renal Nutrition of the National Kidney Foundation. 2005;15(1):125–30. . 15648021 10.1053/j.jrn.2004.09.036

[3] Berg AH, Scherer PE. Adipose tissue, inflammation, and cardiovascular disease. Circulation research. 2005;96(9):939–49. 10.1161/01.RES.0000163635.62927.34 . 15890981

[4] Kalantar-Zadeh K, Kopple JD. Body mass index and risk for end-stage renal disease. Annals of internal medicine. 2006;144(9):701; author reply -2. Epub 2006/05/04. . 16670146 10.7326/0003-4819-144-9-200605020-00021

[5] Segev DL, Simpkins CE, Thompson RE, Locke JE, Warren DS, Montgomery RA. Obesity Impacts Access to Kidney Transplantation. Journal of the American Society of Nephrology. 2008;19(2):349–55. 10.1681/asn.2007050610 18094366 PMC2396750

[6] Chicago UoIa. First simultaneous robotic kidney transplant, sleeve gastrectomy performed. sciencedailycom. 2012, August 31.

[7] Gill JS, Lan J, Dong J, Rose C, Hendren E, Johnston O, et al The Survival Benefit of Kidney Transplantation in Obese Patients. American Journal of Transplantation. 2013;13(8):2083–90. 10.1111/ajt.12331 23890325

[8] Gore JL, Pham PT, Danovitch GM, Wilkinson AH, Rosenthal JT, Lipshutz GS, et al Obesity and Outcome Following Renal Transplantation. American Journal of Transplantation. 2006;6(2):357–63. 10.1111/j.1600-6143.2005.01198.x 16426321

[9] Nicoletto BB F N, Manfro RC, Gonçalves LF, Leitão CB, Souza GC. Effects of Obesity on Kidney Transplantation Outcomes: A Systematic Review and Meta-Analysis. Transplantation. 2014. 10.1097/TP.0000000000000028 24911038

[10] Fontana L, Eagon JC, Trujillo ME, Scherer PE, Klein S. Visceral Fat Adipokine Secretion Is Associated With Systemic Inflammation in Obese Humans. Diabetes. 2007;56(4):1010–3. 10.2337/db06-1656 17287468

[11] PD A. Survival analysis using SAS. A practical guide. SAS Institute Inc 1995.

[12] Liu LL, Lawrence JM, Davis C, Liese AD, Pettitt DJ, Pihoker C, et al Prevalence of overweight and obesity in youth with diabetes in USA: the SEARCH for Diabetes in Youth study. Pediatric diabetes. 2010;11(1):4–11. Epub 2009/05/29. 10.1111/j.1399-5448.2009.00519.x 19473302

[13] Friedman AN, Miskulin DC, Rosenberg IH, Levey AS. Demographics and trends in overweight and obesity in patients at time of kidney transplantation. American Journal of Kidney Diseases. 2003;41(2):480–7. 10.1053/ajkd.2003.50059. 12552513

[14] Ditonno P, Lucarelli G, Impedovo SV, Spilotros M, Grandaliano G, Selvaggi FP, et al Obesity in Kidney Transplantation Affects Renal Function But Not Graft and Patient Survival. Transplantation Proceedings. 43(1):367–72. 10.1016/j.transproceed.2010.12.022 21335224

[15] Hill CJ, Courtney AE, Cardwell CR, Maxwell AP, Lucarelli G, Veroux M, et al Recipient obesity and outcomes after kidney transplantation: a systematic review and meta-analysis. Nephrology Dialysis Transplantation. 2015 10.1093/ndt/gfv214 26044837

[16] Ortiz J, Gregg A, Wen X, Karipineni F, Kayler LK. Impact of donor obesity and donation after cardiac death on outcomes after kidney transplantation. Clinical Transplantation. 2012;26(3):E284–E92. 10.1111/j.1399-0012.2012.01649.x 22686952

[17] Hariharan S, Johnson CP, Bresnahan BA, Taranto SE, McIntosh MJ, Stablein D. Improved graft survival after renal transplantation in the United States, 1988 to 1996. The New England journal of medicine. 2000;342(9):605–12. 10.1056/NEJM200003023420901 . 10699159

[18] Vincenti F, Jensik SC, Filo RS, Miller J, Pirsch J. A long-term comparison of tacrolimus (FK506) and cyclosporine in kidney transplantation: evidence for improved allograft survival at five years. Transplantation. 2002;73(5):775–82. Epub 2002/03/22. . 11907427 10.1097/00007890-200203150-00021

[19] Meier-Kriesche HU, Schold JD, Srinivas TR, Kaplan B. Lack of improvement in renal allograft survival despite a marked decrease in acute rejection rates over the most recent era. American journal of transplantation: official journal of the American Society of Transplantation and the American Society of Transplant Surgeons. 2004;4(3):378–83. Epub 2004/02/14. . 14961990 10.1111/j.1600-6143.2004.00332.x

[20] Leavey SF, McCullough K, Hecking E, Goodkin D, Port FK, Young EW. Body mass index and mortality in ‘healthier’ as compared with ‘sicker’ haemodialysis patients: results from the Dialysis Outcomes and Practice Patterns Study (DOPPS). Nephrology Dialysis Transplantation. 2001;16(12):2386–94. 10.1093/ndt/16.12.2386 11733631

[21] Port FK, Ashby VB, Dhingra RK, Roys EC, Wolfe RA. Dialysis Dose and Body Mass Index Are Strongly Associated with Survival in Hemodialysis Patients. Journal of the American Society of Nephrology. 2002;13(4):1061–6. 11912267 10.1681/ASN.V1341061

[22] Tilg H, Moschen AR. Adipocytokines: mediators linking adipose tissue, inflammation and immunity. Nat Rev Immunol. 2006;6(10):772–83. 10.1038/nri1937 16998510

[23] Kawasaki N, Asada R, Saito A, Kanemoto S, Imaizumi K. Obesity-induced endoplasmic reticulum stress causes chronic inflammation in adipose tissue. Sci Rep. 2012;2 http://www.nature.com/srep/2012/121112/srep00799/abs/srep00799.html#supplementary-information. 10.1038/srep00799 PMC3495279 23150771

[24] Fujioka K. Benefits of moderate weight loss in patients with type 2 diabetes. Diabetes, Obesity and Metabolism. 2010;12(3):186–94. 10.1111/j.1463-1326.2009.01155.x 20151995

[25] de las Fuentes L, Waggoner AD, Mohammed BS, Stein RI, Miller BV Iii, Foster GD, et al Effect of Moderate Diet-Induced Weight Loss and Weight Regain on Cardiovascular Structure and Function. Journal of the American College of Cardiology. 54(25):2376–81. 10.1016/j.jacc.2009.07.054. 20082927 PMC2818984

[26] Blackburn GL, Wollner S, Heymsfield SB. Lifestyle interventions for the treatment of class III obesity: a primary target for nutrition medicine in the obesity epidemic. Am J Clin Nutr. 2010;91(1):289s–92s. Epub 2009/11/13. 10.3945/ajcn.2009.28473D ; PubMed Central PMCID: PMCPmc3131844. 19906805 PMC3131844

[27] Kucharska AM, Pyrżak B, Demkow U. Regulatory T Cells in Obesity In: Pokorski M, editor. Noncommunicable Diseases. Cham: Springer International Publishing; 2015 p. 35–40.

[28] Bouaziz J-D, Yanaba K, Tedder TF. Regulatory B cells as inhibitors of immune responses and inflammation. Immunological Reviews. 2008;224(1):201–14. 10.1111/j.1600-065X.2008.00661.x 18759928

[29] Gerriets VA, MacIver NJ. Role of T Cells in Malnutrition and Obesity. Frontiers in Immunology. 2014;5(379). 10.3389/fimmu.2014.00379 25157251 PMC4127479

[30] Seijkens T, Kusters P, Chatzigeorgiou A, Chavakis T, Lutgens E. Immune Cell Crosstalk in Obesity: A Key Role for Costimulation? Diabetes. 2014;63(12):3982–91. 10.2337/db14-0272 25414012

[31] Ghanim H, Aljada A, Hofmeyer D, Syed T, Mohanty P, Dandona P. Circulating Mononuclear Cells in the Obese Are in a Proinflammatory State. Circulation. 2004;110(12):1564–71. 10.1161/01.cir.0000142055.53122.fa 15364812

