# Supplementary figures and images for: Effect of the Obesity Epidemic on Kidney Transplantation: Obesity Is Independent of Diabetes as a Risk Factor for Adverse Renal Transplant Outcomes
Source: PLoS One. 2016 Nov 16;11(11):e0165712. doi: 10.1371/journal.pone.0165712 (PMC5112887; doi:10.1371/journal.pone.0165712)

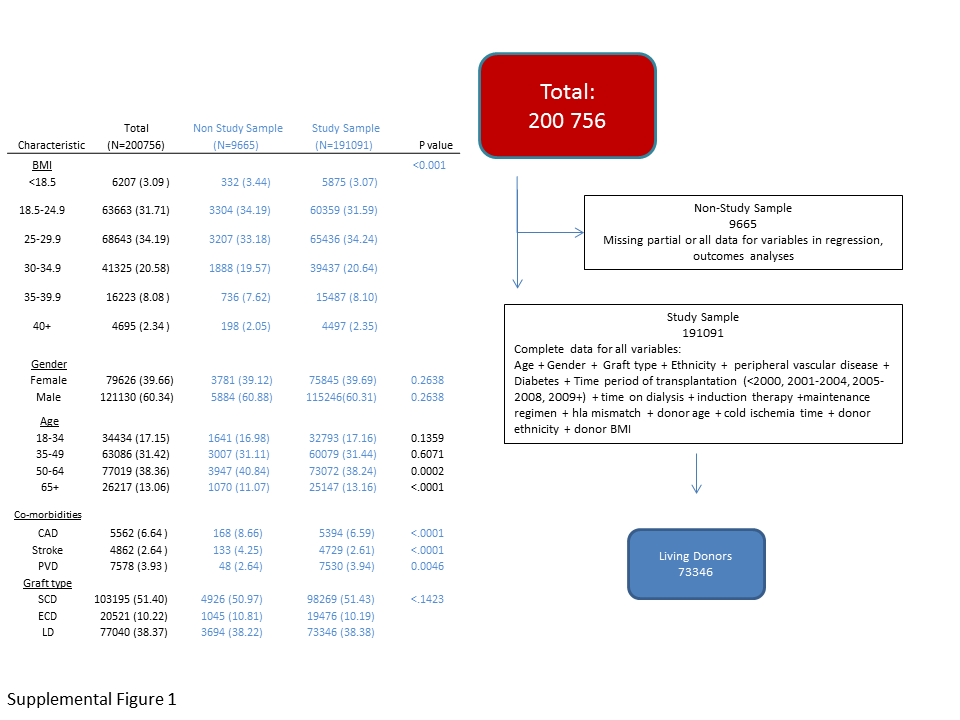

Supplement: S1 Fig — 200756 patients were filtered to identify the study sample, which included pts that had complete data or noted variables. Patients with missing data were excluded. From the patients with complete data, living donor recipients were identified for comparisons to all donors. (TIF) [file pone.0165712.s001.TIF]

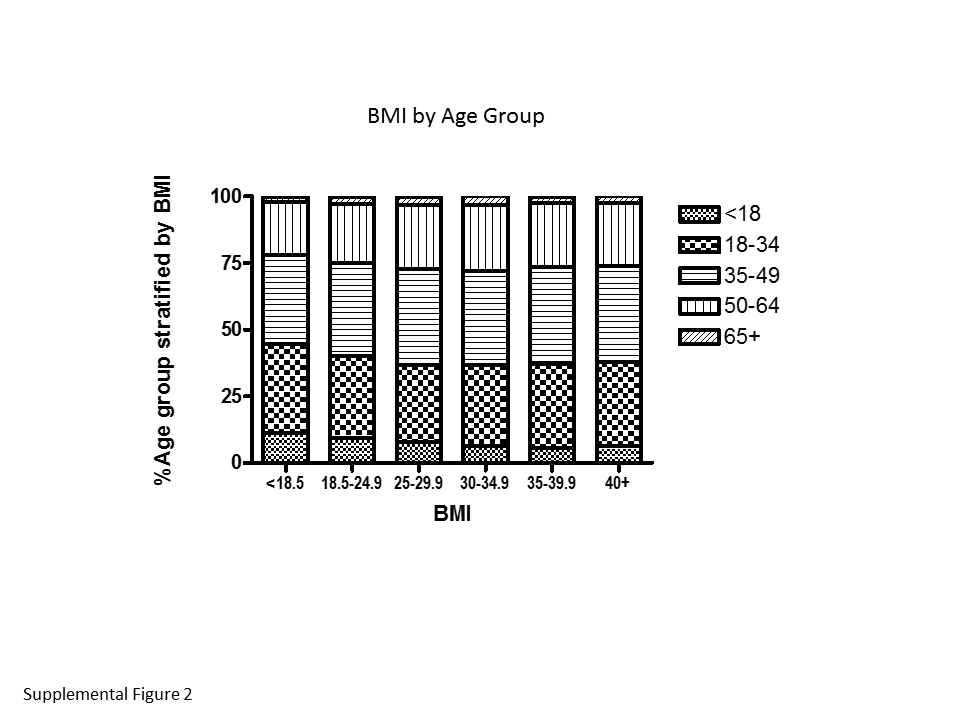

Supplement: S2 Fig — (TIF) [file pone.0165712.s002.TIF]

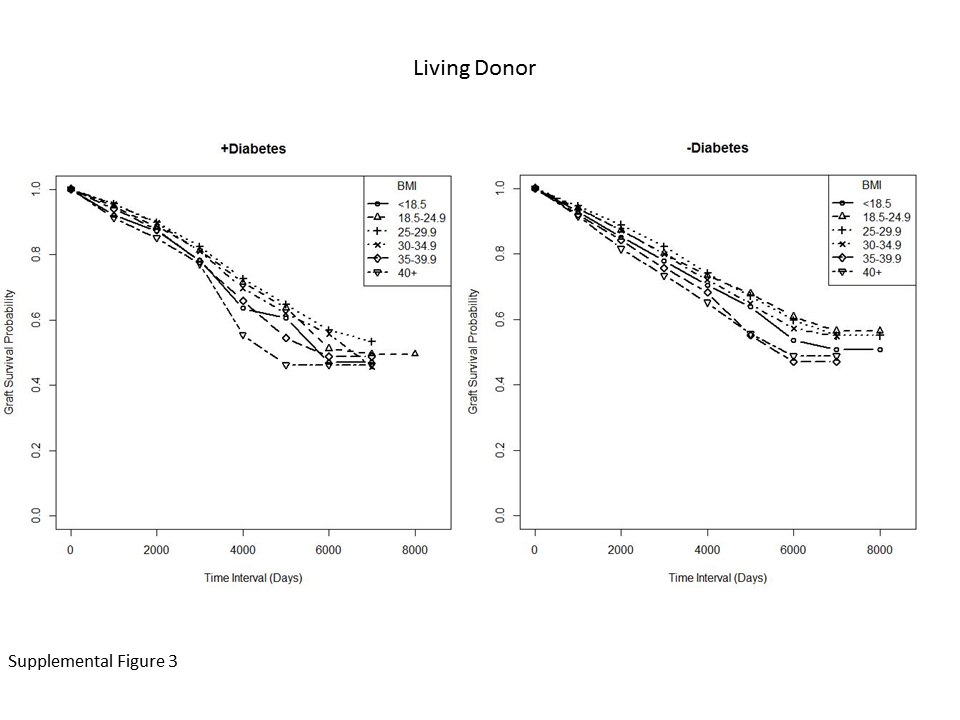

Supplement: S3 Fig — Life-table survival curves were plotted to describe the time-to-failure of those with diabetes (+diabetes) and those without diabetes (-Diabetes) for each BMI category of the living donor recipient cohort. time to failure was restricted over a time course spanning 0 (time of transplant) to 8000 days (21.9 years) with renal transplant patients stratified by BMI is shown. (TIF) [file pone.0165712.s003.TIF]

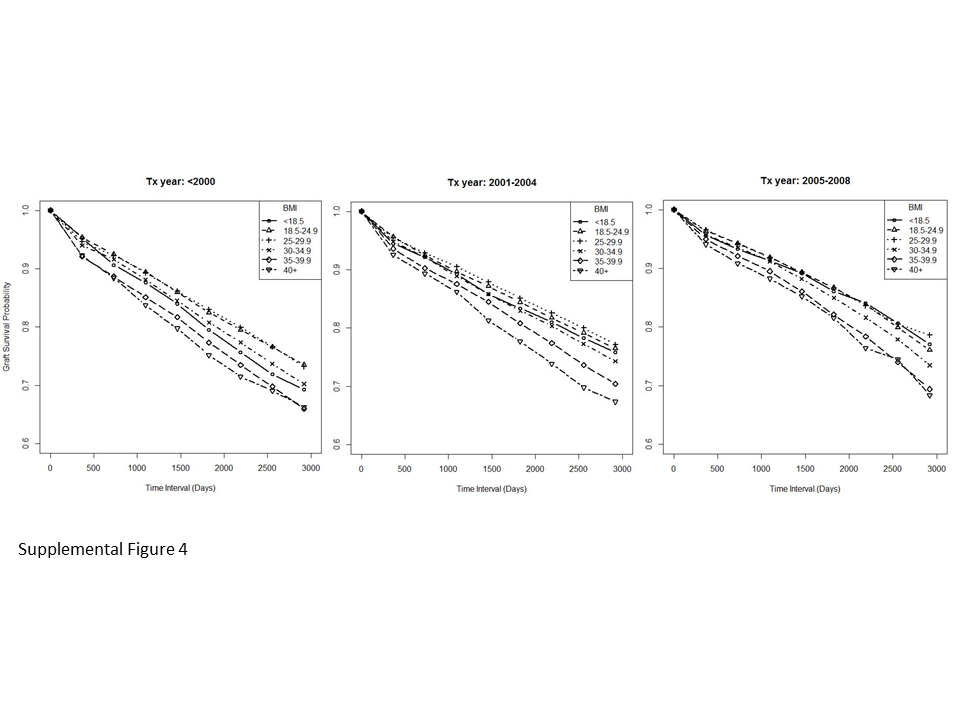

Supplement: S4 Fig — Life-table survival curves were plotted to describe the time-to-failure for each BMI category for all recipients for each indicated time period of transplantation. p<0.05 is significant. (TIF) [file pone.0165712.s004.TIF]

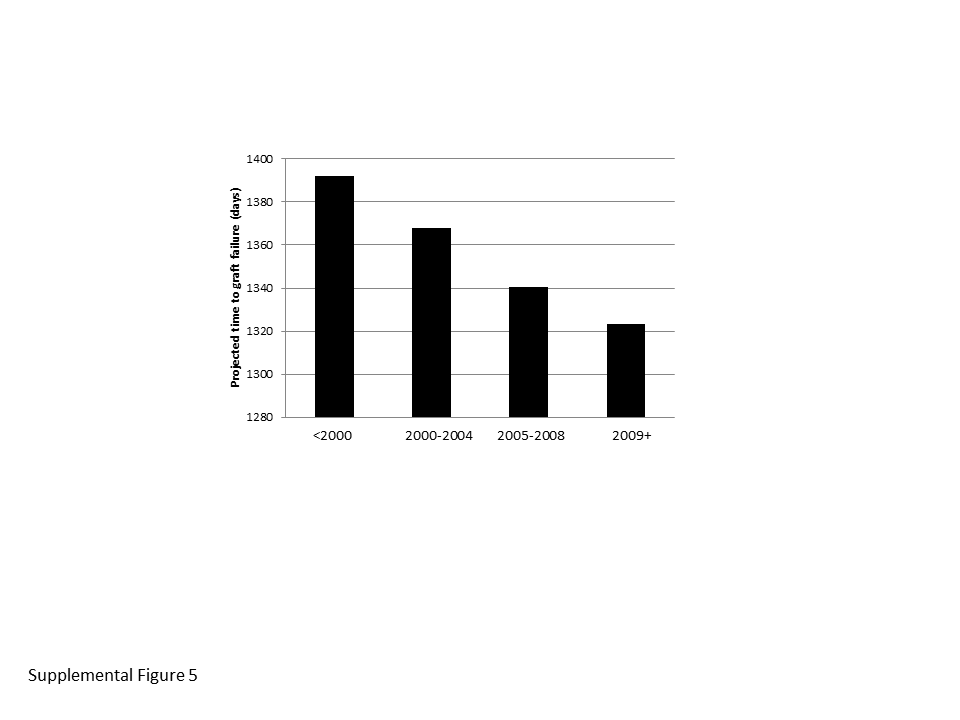

Supplement: S5 Fig — Using the actual number of recipients for each BMI class for each time period (<2000, 2001–2004, 2005–2008, 2009+), we projected that time to graft failure assuming all other variables were constant. (TIF) [file pone.0165712.s005.TIF]

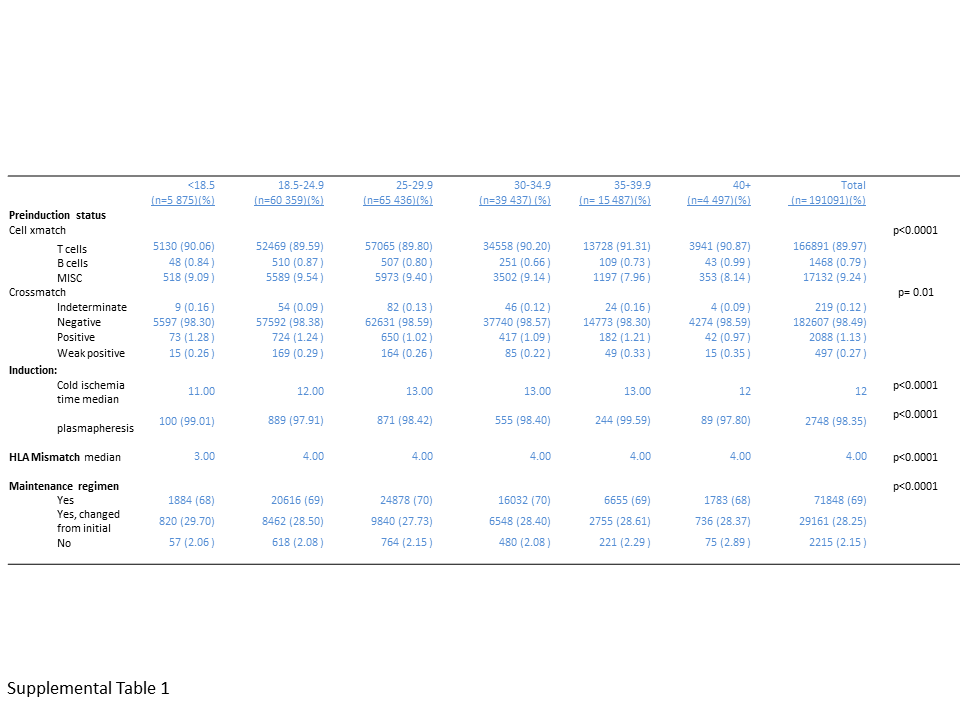

Supplement: S1 Table — (TIF) [file pone.0165712.s006.TIF]
